# Our Work Here and Hereafter

**Published:** 1918-04-13

**Authors:** Dean Stubbs


### Our Work Here and Hereafter.

**And for the Dead of Death, to Thee**
**I trust it; for indeed I know that he**
**Who through his life's appointed days**
**Has stood not idle in the market-place,**
**He dies not, no! there is no death for him,**
**No death, but only change**
**Beyond this earthly range;**
**New life, new work, with servant seraphim,**
**O Lord of Service! Lord of Life!**
**Grant me that guerdon in the other life,**
**New service there—that with my latest breath**
**Be my one prayer, O living Lord of Death!**—Dean Stubbs.

Previous articles appeared Dec. 8, 15, 22, Jan. 5, 12, 26, Feb. 2, March 2, 9, 23 and 30, p. 555.

